# Patient-specific pulse wave propagation model identifies cardiovascular risk characteristics in hemodialysis patients

**DOI:** 10.1371/journal.pcbi.1006417

**Published:** 2018-09-14

**Authors:** Jan Poleszczuk, Malgorzata Debowska, Wojciech Dabrowski, Alicja Wojcik-Zaluska, Wojciech Zaluska, Jacek Waniewski

**Affiliations:** 1 Department for Mathematical Modeling of Physiological Processes, Nalecz Institute of Biocybernetics and Biomedical Engineering Polish Academy of Sciences, Warsaw, Poland; 2 Department of Anesthesiology and Intensive Therapy, Medical University of Lublin, Lublin, Poland; 3 Department of Physical Therapy and Rehabilitation, Medical University of Lublin, Lublin, Poland; 4 Department of Nephrology, Medical University of Lublin, Lublin, Poland; University of California Irvine, UNITED STATES

## Abstract

Risk of cardiovascular associated death in dialysis patients is the highest among all other co-morbidities. Improving the identification of patients with the highest cardiovascular risk to design an adequate treatment is, therefore, of utmost importance. There are several non-invasive cardiovascular state biomarkers based on the pulse (pressure) wave propagation properties, but their major determinants are not fully understood. In the current study we aimed to provide a framework to precisely dissect the information available in non-invasively recorded pulse wave in hemodialysis patients. Radial pressure wave profiles were recorded before, during and after two independent hemodialysis sessions in 35 anuric prevalent hemodialysis patients and once in a group of 32 healthy volunteers. Each recording was used to estimate six subject-specific parameters of pulse wave propagation model. Pressure profiles were also analyzed using SphygmoCor software (AtCor Medical, Australia) to derive values of already established biomarkers, i.e. augmentation index and sub-endocardial viability ratio (SEVR). Data preprocessing using propensity score matching allowed to compare hemodialysis and healthy groups. Augmentation index remained on average stable at 142 ± 28% during dialysis and had similar values in both considered groups. SEVR, whose pre-dialytic value was on average lower by 12% compared to healthy participants, was improved by hemodialysis, with post-dialytic values indistinguishable from those in healthy population (p-value > 0.2). The model, however, identified that the patients on hemodialysis had significantly increased stiffness of both large and small arteries compared to healthy counterparts (> 60% before dialysis with p-value < 0.05 or borderline) and that it was only transiently decreased during hemodialysis session. Additionally, correlation-based clustering revealed that augmentation index reflects the shape of heart ejection profile and SEVR is associated with stiffness of larger arteries. Patient-specific pulse wave propagation modeling coupled with radial pressure profile recording correctly identified increased arterial stiffness in hemodialysis patients, while regular pulse wave analysis based biomarkers failed to show significant differences. Further model testing in larger populations and investigating other biomarkers are needed to confirm these findings.

## Author summary

There are more than 2 million people receiving hemodialysis (HD) treatment worldwide. Cardiovascular disease is the most common cause of death in those patients. There are several non-invasive methods to assess if a person from general population has a high risk for developing cardiovascular disease, but it is unclear whether they are useful in hemodialysis patients. Here we assessed the ability of patient-specific pulse wave propagation modeling to correctly identify high cardiovascular risk factors in hemodialysis patients. We performed pulse wave analysis (PWA) in patients on hemodialysis and in healthy subjects. Recorded peripheral pressure profiles were simultaneously used to inform subject-specific mathematical model of pulse wave propagation. We found that standard PWA-derived biomarkers failed to clearly show the differences between hemodialysis patients and healthy subjects. However, proposed mathematical model of pulse wave propagation identified significantly increased arterial stiffness in HD patients and provided also the major determinants of PWA-derived biomarkers. Our study suggests that current pulse wave analysis based biomarkers can be insufficient to accurately diagnose hemodialysis patients. Proposed patient-specific pulse wave propagation modeling framework may be a new tool to assess the cardiovascular risk in both general and hemodialysis populations.

## Introduction

Chronic kidney disease (CKD) is associated with a significant increase in cardiovascular disease (CVD) incidence, with almost two-fold increase of CVD prevalence in elderly CKD patients in US [1]. At the same time, death due to the cardiovascular diseases, with congestive heart failure and peripheral arterial disease being the two most common, is the single largest cause of attributable mortality in the prevalent and incident dialysis patients [1]. Thus, the development of sensitive and non-invasive cardiovascular diagnostic methods is of great importance for patients with more severe forms of chronic kidney disease, as they could be used to design better treatment.

High incidence of cardiovascular diseases in dialysis patients is related, among others, to high prevalence of vascular calcification caused by abnormal mineral metabolism that yields increased arterial wall stiffness [2–4]. Pulse wave velocity (PWV) measurement is the gold-standard method for the assessment of arterial stiffness [5, 6] with higher PWV values indicating less elastic vessels. Most of the commercial devices dedicated to PWV estimation rely on simultaneous gating of electrocardiograph with pressure waveform recordings in two separate peripheral vessels (typically carotid and femoral) [7, 8]. However, this electromechanical approach can bring a large discomfort to a patient [9], takes a substantial amount of time to perform the measurement, and relies on external measurements of distances between the pulse wave recording sites what is only a coarse grain approximation of the true artery lengths.

**Fig 1:**
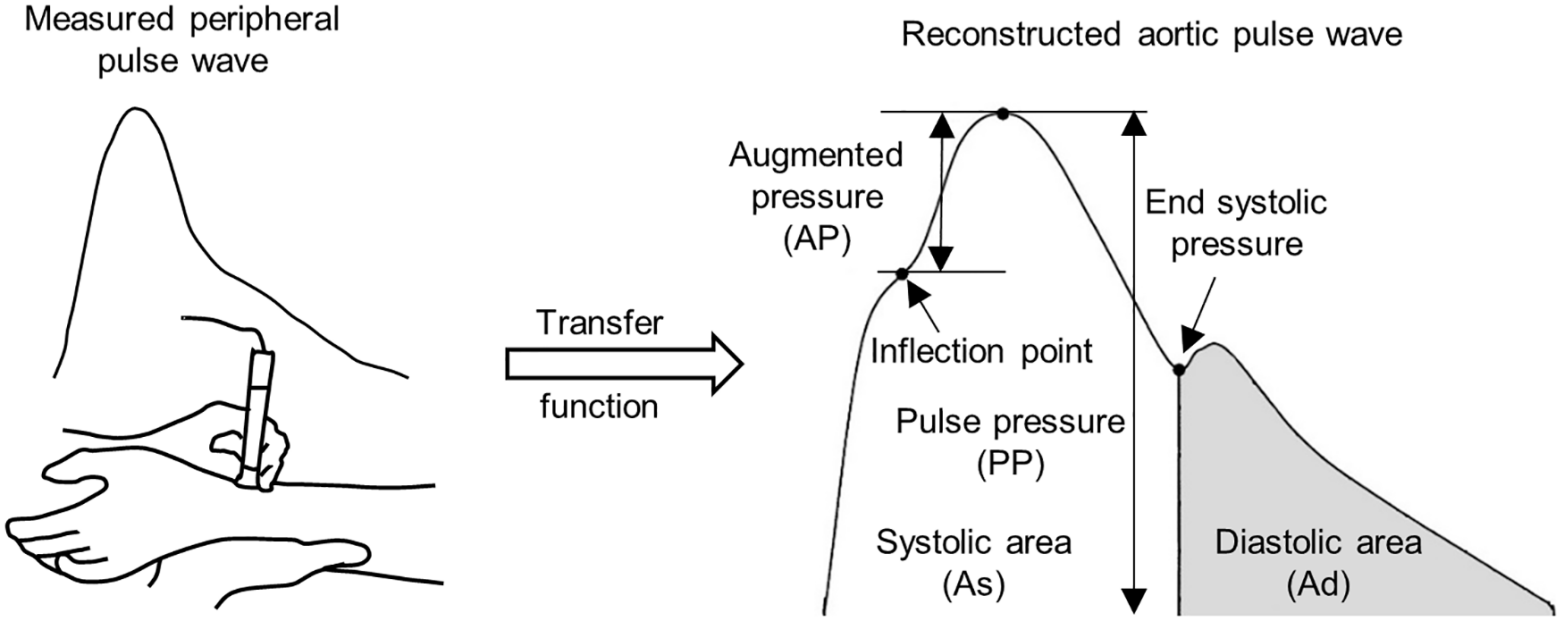
Pulse wave analysis technique. Non-invasive pulse wave recording in peripheral artery is used to synthesize the aortic pressure wave using a generalized transfer function. Characteristic points in the aortic pressure waveform are then used to calculate various indices related to the cardiovascular system state.

Pulse wave analysis (PWA) is another and much simpler to perform non-invasive technique that provides various cardiovascular system state indices based on a peripheral pulse (pressure) wave recording [10, 11]; see Fig 1. The underlying assumption of the PWA method is that there exists a generalized transfer function that allows to reconstruct the aortic pulse waveform from the peripheral pressure recording for any individual from general population [10, 11]. However, despite some studies showing validity of this assumption [12–14], there are still significant concerns about the robustness of aortic wave reconstruction [15–19]. Moreover, it is not completely clear what the major determinants of PWA-derived indices are as the waveform can be attributed to those cardiovascular properties that determine the wave propagation and reflection, such as, among other, arterial geometry, elasticity of the vessel wall, systemic resistance, heart frequency, cardiac output [20]. Nevertheless, one of the PWA-derived indices, i.e. augmentation index (AI = PP/(PP-AP)x100%; see Fig 1) that represents the augmentation of central pressure height that is being introduced by the reflected waves [21], has been clearly associated with aging and cardiovascular risk [22–25].

Here, in a detailed study of pulse wave propagation properties using our previously published model [20], we aimed to check whether the cardiovascular risk factors for hemodialysis patients, such as arterial stiffness, can be derived from a single, quick and non-invasive peripheral wave recording and thus, overcome the PWA and PWV limitations. Moreover, we try to decipher what are the major determinants of PWA-derived indices and how they relate to the predicted pulse wave velocity.

## Methods

### Ethics statement

The study was approved by the Bioethical Committee at the Medical University of Lublin (Poland) and informed consent has been obtained from all patients.

### Study subjects

Two standard hemodialysis (HD) sessions (duration 240.2 ± 13.4 min) were monitored in 35 anuric, prevalent hemodialysis patients (dialysis vintage 9.1 ± 8.9 years). Basic patients’ characteristics are shown in Table 1. At the time of the study none of the patients had diagnosed cardiovascular disease. All patients underwent their regular treatment with ultrafiltration volume set to achieve patient-specific post-dialytic dry weight (ultrafiltration rate of 11 ± 3.6 ml/min). The average flow of blood and dialysis fluid in extracorporeal circuit was 287.3 ± 47.4 ml/min (range 180 − 380 ml/min) and 500 ml/min, respectively. All patients had arteriovenous fistulas. We enrolled an additional group of 32 healthy volunteers (control group) in order to investigate the differences between HD and general population. The only enrollment criterion for this group was lack of diagnosed cardiovascular disease. Basic volunteers characteristics are shown in Table 1. The data about 20 out of those 32 healthy individuals have been published previously in [20]. Participants were enrolled from June 2014 through February 2018.

**Table 1:** Basic hemodialysis (HD) and control groups characteristics before and after age and gender based propensity score matching (PSM). Reported values for HD group were assessed after midweek hemodialysis session. The data about 20 out of 32 control subjects have been published previously in [20].

	All subjects		After PSM	
	HD	Control	HD	Control
	(N = 35)	(N = 32)	(N = 18)	(N = 29)
*Gender, % male*	43%	47%	39%	41%
*Age, years*	61.2 ± 14.3	42.7 ± 9.3***	49.6 ± 8.1	44.3 ± 8.3
*Height, cm*	167.9 ± 9.4	173.4 ± 10.8	168.6 ± 8.8	173.4 ± 11.3
*Weight, kg*	72.2 ± 19.9	78.3 ± 19.1	72.6 ± 24.3	78.4 ± 19.6
*BMI, kg/m^2^*	25.4 ± 5.6	25.7 ± 3.8	25.3 ± 7.3	25.7 ± 3.8

***significantly different than for HD group; p − value < 0.001; Wilcoxon-test

In order to compare measurements and model predictions between the HD and control groups we preprocessed the data using age and gender based propensity score matching (PSM) technique [26]. This approach allowed us to select the subgroups for which there were no statistically significant differences when comparing basic characteristics; compare last two columns in Table 1.

### Pulse wave analysis

**Fig 2:**
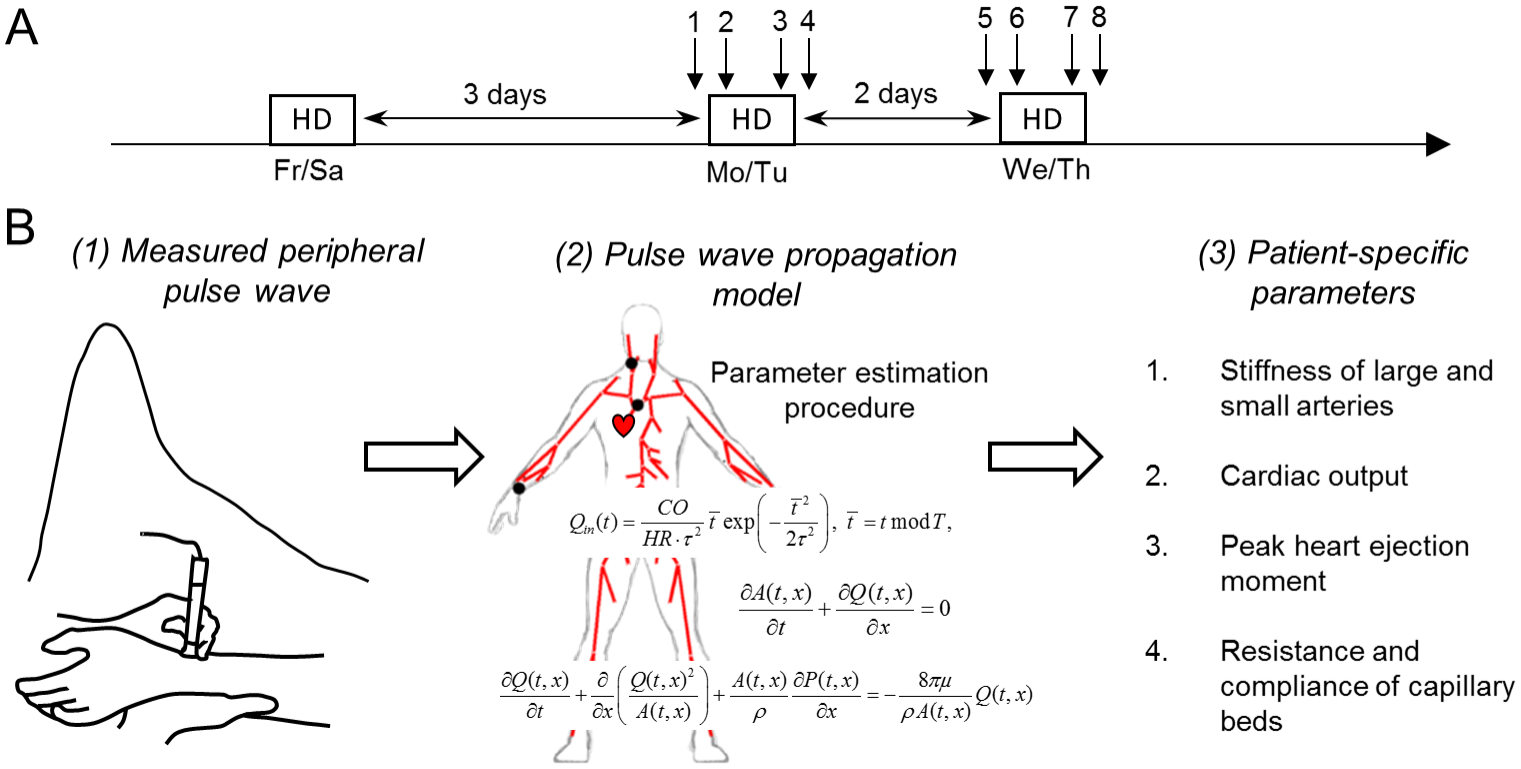
Study workflow. (A) Eight separate recordings of radial pulse wave were performed for each dialysis patient. In healthy individuals recording was performed only once. (B) Each radial pressure recording was used to estimate parameters of the pulse wave propagation model and derive patient-specific information about cardiovascular system state.

Pulse wave shape in radial artery was recorded using applanation tonometry (SphygmoCor, AtCor Medical, Australia) once in healthy individuals and about 15 minutes before start, after start, before end, and after end of two hemodialysis sessions performed after 3- and 2-day interdialytic intervals; see Fig 2A. All measurements were made in at least duplicate and the recording with higher quality (defined and calculated by SphygmoCor software as ‘operator index’) was chosen. Measurements with insufficient quality (’operator index’ ≤ 74) were excluded in accordance to the user manual. All recordings were performed by one trained clinician with measurement performed in the non-fistula arm in the case of hemodialysis patients. The radial pulse wave was calibrated to the blood pressure measured oscillometrically at the brachial artery (Omron M3, Omron Healthcare, Kyoto, Japan). We used SphygmoCor software together with its built-in transfer function to derive systolic (SP) and diastolic (DP) pressures in ascending aorta, together with the augmented pressure (AP) which is the additional pressure added by the reflected wave to the forward pressure wave. We have extracted also augmentation index (AI = (SP-DP)/(SP-DP-AP)x100%) and ejection duration (ED) being the time from the start of the pulse to the end of systole. Information about ejection duration is used by the SphygmoCor software to calculate sub-endocardial viability ratio (SEVR) which serves as an estimate for the adequacy of myocardial blood flow.

### Pulse wave propagation model

We use our previously published model [20] that allows to simulate the blood flow in a bifurcating binary tree of fifty-five larger systemic arteries in which individual vessels are axisymmetric elastic cylinders tapering along their length. The model describes spatiotemporal changes in the pressure and blood flow, under the assumption that each vessel is impermeable and blood is an incompressible fluid with given density and viscosity. The model is formulated as a large system of partial differential equations coupled to a series of outflow conditions expressed using ordinary differential equations.

Here, as in [20], we use the model to estimate six patient-specific parameters: *k*_1_ and *k*_3_ being hyperparameters describing stiffness of small and large arteries, respectively; cardiac output (CO); moment of the heart ejection peak (*τ*); and scaling constants of resistances (*S*_*R*_) and compliances (*S*_*C*_) of capillary beds (Fig 2B). Equation describing stiffness of an artery (*F*) has the following form

$$\begin{array}{c} F\left(x\right)=\frac{4}{3}(k_{1}\exp\left(-k_{2}r_{0}\left(x\right)\right)+k_{3})\;, \end{array}$$

where *k*_2_ = 22.53 and *r*_0_(*x*) is the assumed vessel diameter at the reference pressure (97 mmHg) and distance *x* from the vessel’s inlet [20, 27]. Therefore, larger vessels’ (*r*_0_ > > 1) stiffness is governed primarily by parameter *k*_3_ whereas for smaller vessels (*r*_0_ < < 1) stiffness *F* is related to *k*_1_ + *k*_3_. Certain amount of the inter-patient variability in *r*_0_ is introduced through scaling of the nominal arterial tree definition according to the patients’ height; see [20] for further details.

Parameters *S*_*C*_ and *S*_*R*_ are used to respectively scale nominal compliances and resistances in the Windkessel models imposed at each terminal end of the modeled arterial tree. The goal of the parameter estimation procedure was to find model parameters for which model-predicted radial pressure waveforms correspond the best to those recorded using applanation tonometry; see [20] for the details and other fixed parameters values. Most importantly, after calibrating the model parameters using patient-specific radial pressure waveform recording we can easily compute patient-specific pulse wave velocity between any two given points within the modeled arterial tree.

### Statistical analyses

The data are presented as mean ± standard deviation (SD) and statistical significance was set at the level of p-value = 0.05, unless otherwise indicated. Statistical dependence between variables was tested using Spearman’s correlation coefficient. Changes in model- and SphygmoCor-derived parameters related to dialysis were investigated by Wittkowski test followed by multiple pairwise comparison analysis based on adjusted Scheffe’s procedure. Wittkowski test is a Friedman-type statistics for consistent multiple comparisons for unbalanced designs with missing data [28]. Due to insufficient radial wave recording quality, there were 25 missing records (among 280) in the HD group database. The Wilcoxon rank-sum test with normal approximation was used to compare continuous factors between hemodialysis and healthy subjects groups. Pearson’s Chi-squared test with Yates’ continuity correction was used to compare categorical factors.

## Results

### Quality of pulse pressure approximation in hemodialysis patients

**Fig 3:**
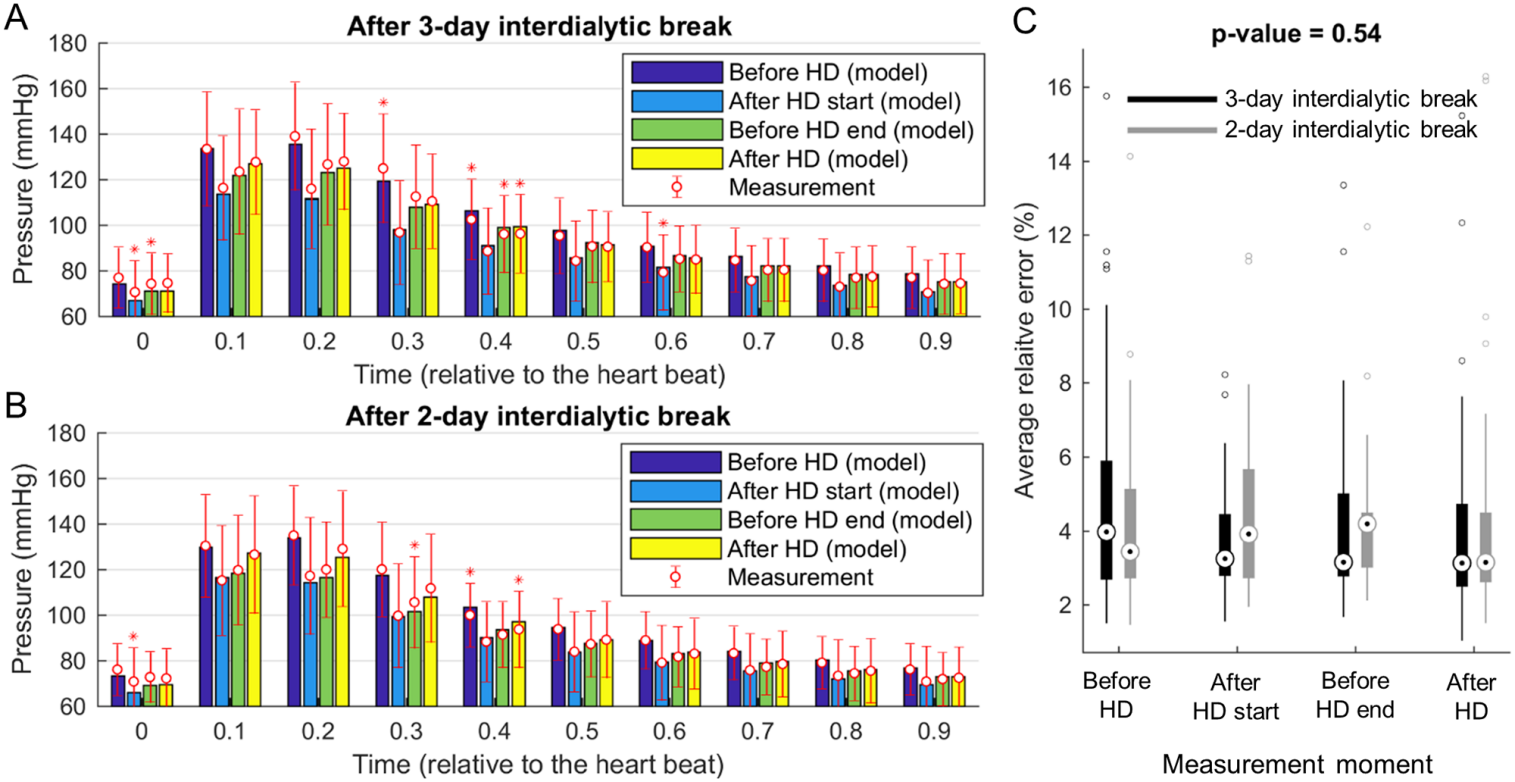
Comparison of the model predicted and measured pressure profiles in radial artery. Bars represent average model-predicted pressures at different measurement moments after 3- (A) and 2-day (B) interdialytic break. Shown are also inter-patient means and standard deviations for measurements (circles with whiskers) whereas standard deviation from the model is omitted for clarity. Asterisks indicate statistical difference at p-value < 0.05 calculated using paired sample t-Test. (C) Quality of the model approximation, i.e. average relative error between simulated and recorded radial pressure waveform, does not depend on the measurement moment.

We have previously shown that the model is able to reproduce clinically measured radial waveforms in healthy individuals [20]. After applying the same data fitting procedure as in [20] we obtained also an excellent agreement between measured and simulated radial pressure profiles in hemodialysis patients, compare Fig 3A and 3B. Average relative error between simulated and recorded pressure profile was about 4% and did not depend on the measurement moment, Fig 3C. The average values of model-estimated patient-specific parameters were: *k*_3_ = 14.51 ± 9.03 x10^5^g/(s^2^ cm), *k*_1_ = 2.28 ± 1.73 x10^7^g/(s^2^ cm), CO = 3.56 ± 0.8 l/min, *τ* = 101.08 ± 20.78 ms, *S*_*C*_ = 13.57 ± 4.34, and *S*_*R*_ = 1.34 ± 0.56. In line with our previous study [20] we found that women had on average lower stroke volume (SV) than men (SV = CO/HR; 51.3 ± 16.4 vs. 54.9 ± 15 ml; p-value = 0.02). In the hemodialysis group, however, we have also found gender differences in the time to the heart ejection peak (*τ*; 92.3 ± 16.2 vs. 108 ± 21.4 ms for males and females, respectively; p-value < 0.001) and resistances of capillary beds (*S*_*R*_; 1.51 ± 0.56 vs. 1.22 ± 0.54 for males and females, respectively; p-value < 0.001).

### Effect of hemodialysis on the model-estimated parameters

**Fig 4:**
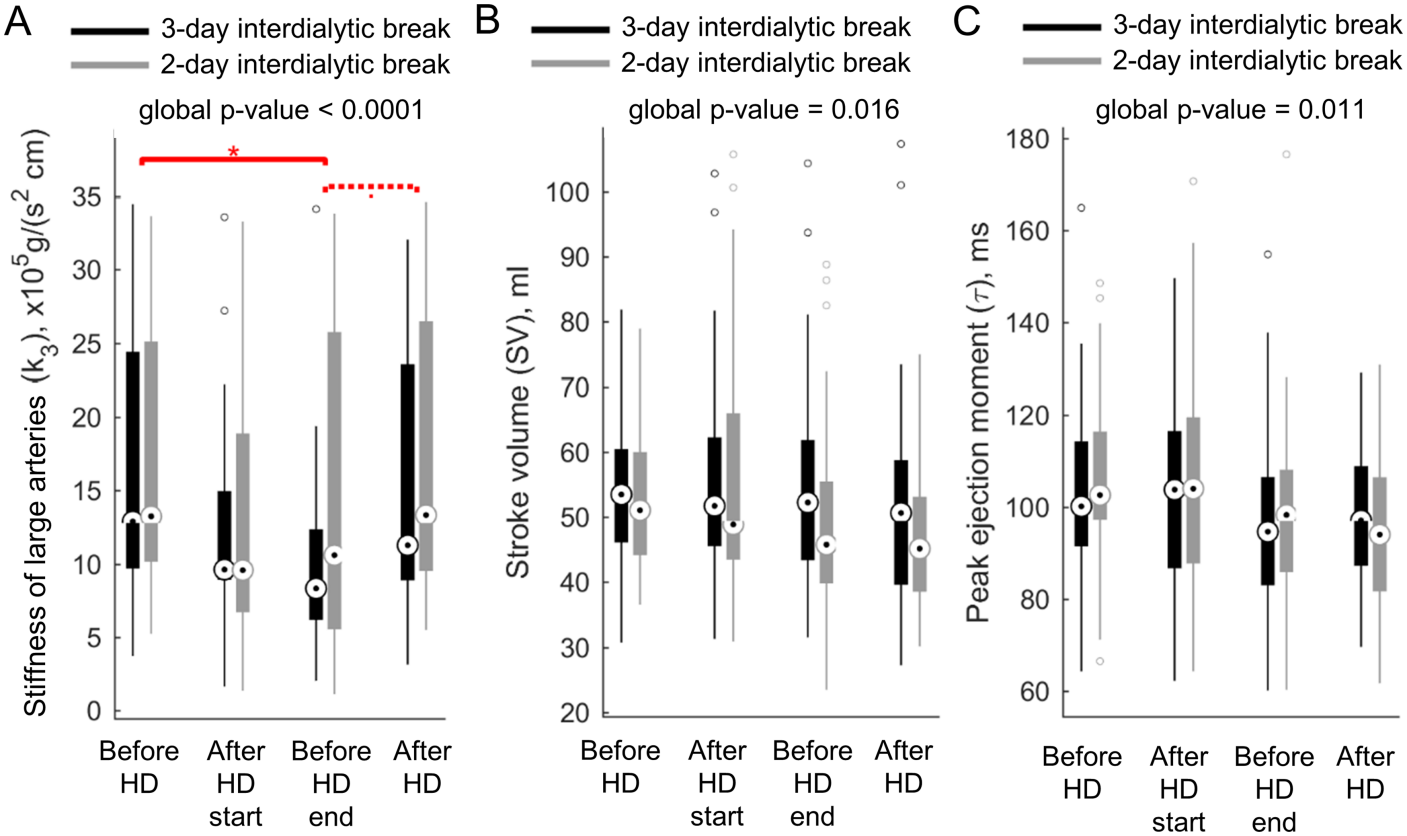
Effects of hemodialysis on the model-estimated parameters. Repeated measurements statistical testing revealed that hemodialysis affects the model-predicted stiffness of large arteries (A), stroke volume (B), and peak heart ejection moment (C). Each box represents the interquartile range with the circle within being the sample median. Hollow circles denote outliers, i.e. values that are more than 1.5 times the interquartile range away from the top or bottom of the box. Whiskers show the furthest observations when neglecting outliers. The global p-value from the Wittkowski test statistics is shown above each boxplot. Solid red line with an asterisk above indicates multiple-comparison p-value < 0.05 whereas dotted line shows borderline statistical significance, i.e. p-value < 0.1.

Hemodialysis had a significant effect on the model-estimated parameter describing stiffness of large arteries (*k*_3_) with the most pronounced decrease from 16.44 ± 8.73 to 9.92 ± 6.81 x10^5^g/(s^2^ cm) when comparing average values before and just before the end of HD session performed after 3-day interdialytic break; compare Fig 4A. This drop in arterial stiffness was directly related to the change of model-predicted pulse wave velocity calculated from the aortic arch to the femoral artery which decreased in the same period from 9.97 ± 2.67 to 7.7 ± 2.46 m/s (p-value = 0.021). Interestingly, those changes were only transient as the pre- and post-dialytic stiffness was not statistically different for both considered interdialytic break lengths. Statistical testing revealed also that hemodialysis has an impact on the model-estimated stroke volume and time to the heart ejection peak (*τ*), but the multiple comparisons testing was not able to provide clear answer about the direction of those hemodialysis induced changes; compare Fig 4B and 4C. From the boxplots, however, it seems that value of both of those parameters decrease during hemodialysis.

### Differences between hemodialysis and control groups

**Fig 5:**
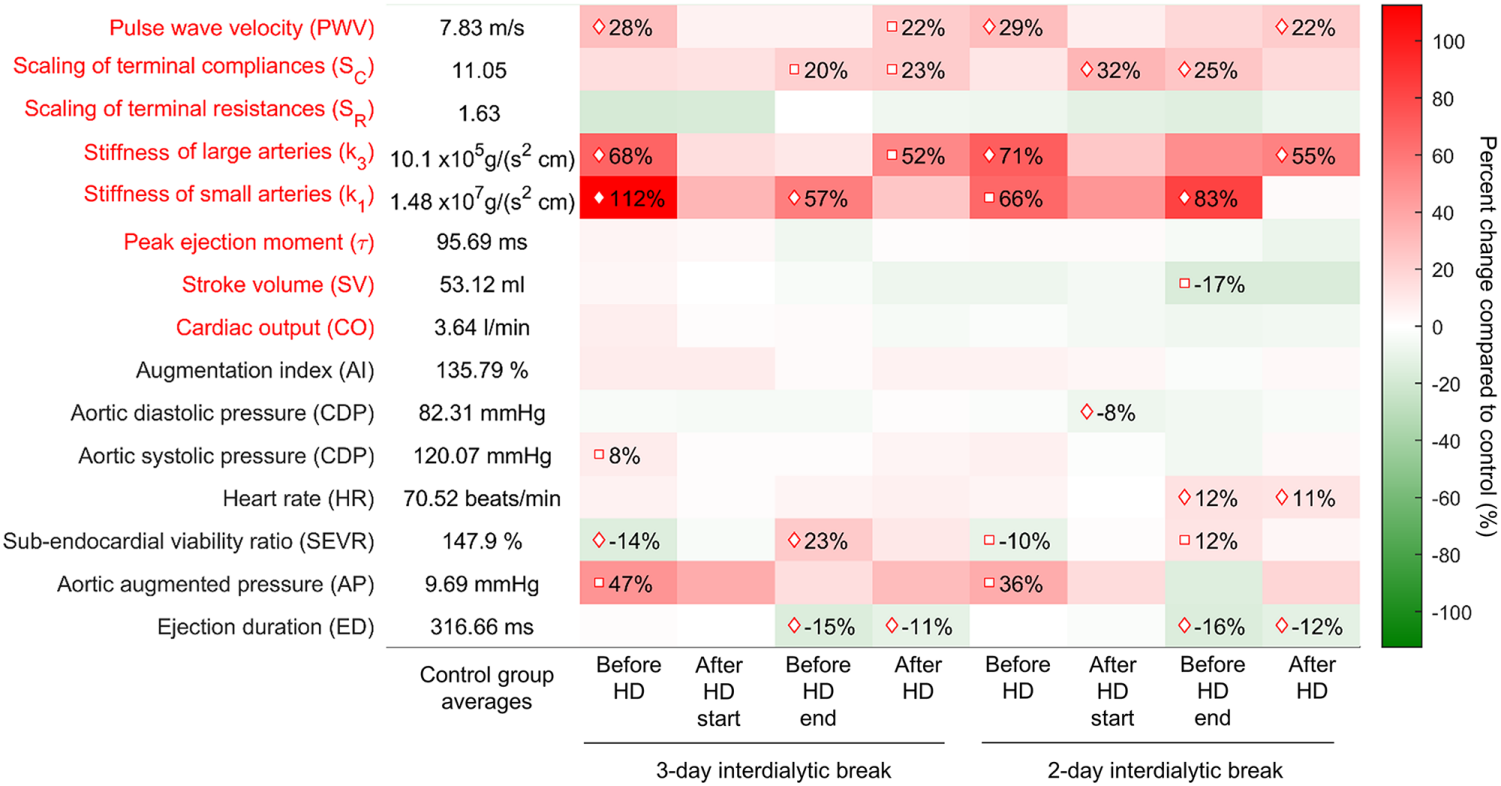
Comparison of the control and hemodialysis (HD) groups after propensity score matching. First column shows average values of the model-estimated (names in red font) and SphygmoCor-derived (names in black font) parameters in control group. Colormap encodes the percent change in the average parameter value for hemodialysis patients compared to healthy counterparts. Exact percent change value is shown in the cases for which the difference is statistically significant (diamond for p-value < 0.05 and square for borderline p-value < 0.1).

Despite the significant differences in the pre-dialytic values of augmented pressure (AP) for both considered interdialytic break lengths, augmentation index (AI) calculated by the SphygmoCor device was not significantly different between propensity score matched groups, with the average value of 135.79 ± % and 142 ± 28% for healthy subjects and hemodialysis patients, respectively; compare Fig 5. Sub-endocardial viability ratio (SEVR), whose pre-dialytic value was on average lower by 12% compared to healthy subjects, was improved by hemodialysis, with post-dialytic values indistinguishable from those in control group (p-value >0.2). Systolic and diastolic aortic blood pressures derived by SphygmoCor showed only minor differences between considered groups with only two moments for which the differences were statistically significant. Patients just before the end and just after hemodialysis had shorter SphygmoCor-calculated ejection duration, but it seems that this change may be directly related to the increase in the heart rate, at least for the measurements performed after 2-day interdialytic break; compare the first and the fourth row from the bottom in Fig 5.

The model, however, clearly identified that the individuals on hemodialysis had significantly increased stiffness of both large and small arteries compared to the healthy counterparts (> 60% difference before dialysis with p-value < 0.05 or borderline) and that the stiffness was only transiently decreased during hemodialysis session; see the fourth and the fifth row from top in Fig 5. Those differences are reflected in significantly larger model-predicted velocities of the pulse waves traveling from the ascending aorta to the femoral artery; see the first row in Fig 5. The model identified also that the patients on hemodialysis have increased compliances of the capillary beds when compared to the healthy individuals, what probably counteracts the blood pressure increase caused by the larger arterial stiffness, because the model-predicted cardiac output remains unchanged.

### Major determinants of pulse wave analysis derived indices

**Fig 6:**
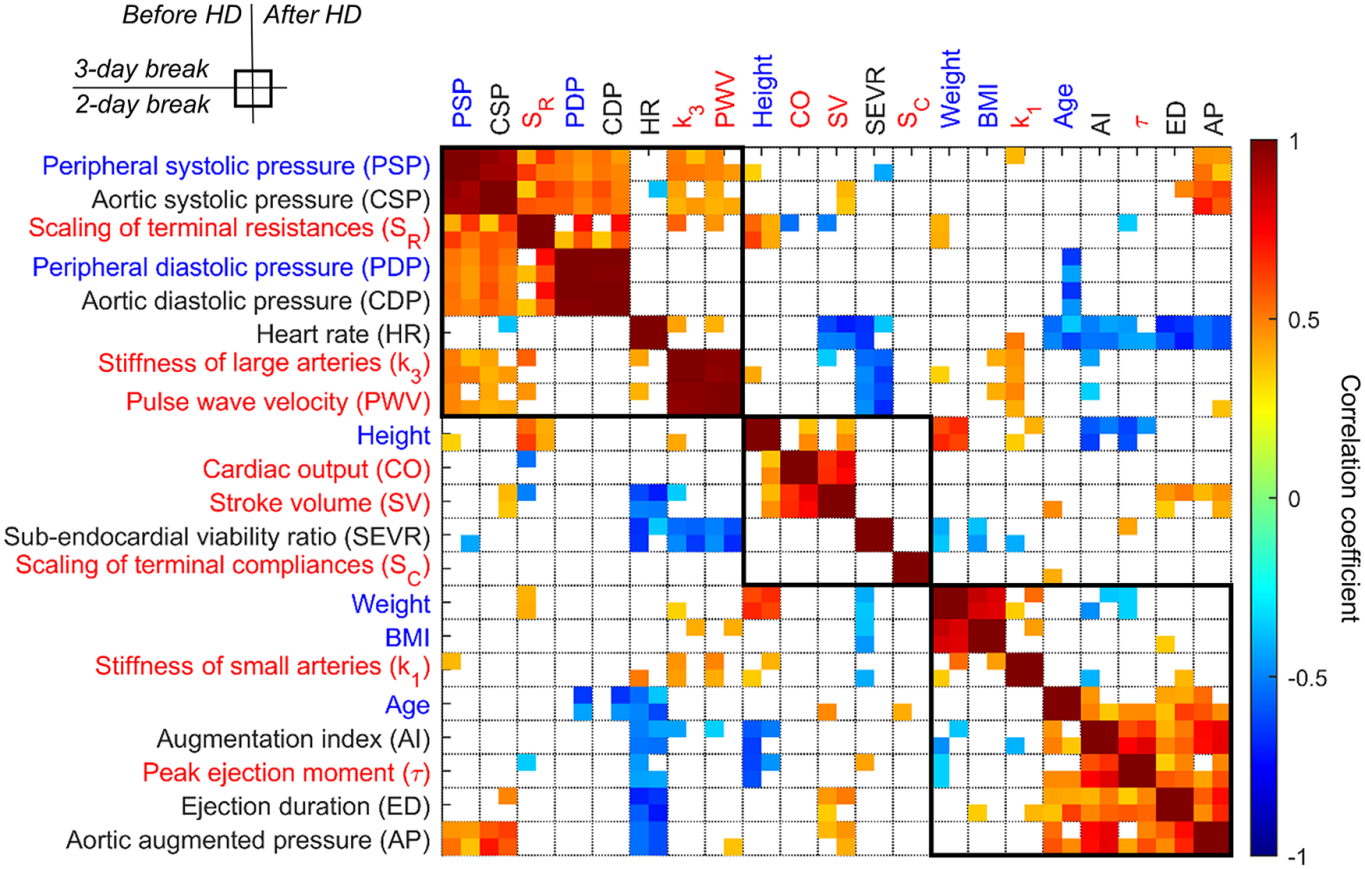
Interdependence of the clinical and pulse wave-derived characteristics in hemodialysis patients. Color coded are the Spearman correlation coefficients for four different pulse wave measurement moments. Font color encodes the type of the parameter, i.e. whether it is model-derived (red), SphygoCor-derived (black), or it is a basic clinical characteristic (blue). White squares indicate lack of statistical significance. Three black squares highlight the main correlation clusters.

In order to detect the major determinants of the standard pulse wave analysis indices, i.e. augmentation index, augmented pressure, and sub-endocardial viability ratio, we calculated their correlation coefficients with the model-estimated parameters and basic clinical characteristics. In line with our previous study [20], correlation based hierarchical clustering revealed that both the augmented pressure and the augmentation index are related to the heart ejection profile, i.e. model-derived peak heart ejection moment and SphygmoCor-derived ejection duration, rather than to the stiffness of arteries, see Fig 6. The strongest positive correlation of SphygmoCor-derived augmentation index, in addition to the obvious dependence on augmented pressure, was detected when comparing its value with the parameter describing the shape of heart ejection profile (*τ*; average R = 0.71; maximal p-value < 0.001), i.e. the steeper is the ejection profile the smaller is the augmentation index.

More closely related to the stiffness of large arteries (parameter *k*_3_) was SphygmoCor-derived SEVR, i.e. it correlated negatively with *k*_3_ value. Interestingly, the correlation analysis revealed that the major determinant of both peripheral and aortic peak pressures is, in addition to the arterial stiffness, the resistance of the capillary beds, see top-left cluster in Fig 6. We found that the above relationships are similar for healthy individuals, see S1 Fig. In the case of healthy individuals, however, SEVR correlated with the model-estimated PWV rather than the stiffness of larger arteries and augmentation index was also negatively correlated with the stiffness of small arteries S1 Fig.

## Discussion

Information about the patient’s cardiovascular system state can be nowadays obtained non-invasively by analyzing pulse waveform recordings from peripheral arteries. Pulse wave analysis method relies on the reconstruction of the aortic blood pressure waveform from the peripheral measurement using generalized transfer function, an approach that has been validated but also questioned by some studies [16, 29–33]. It is also not completely clear what the major determinants of PWA-derived indices are as the waveform can be attributed to multiple cardiovascular properties that determine the wave propagation and reflection properties [20]. Nevertheless, the clinical benefits of using PWA to assess the cardiovascular risk and the impact of therapeutic intervention on the central blood pressure have been clearly shown in clinical trials [34, 35]. Simultaneous gating of the electrocardiographs with peripheral pressure recordings at two distinct sites allows to estimate pulse wave velocity (PWV) which is a validated biomarker for arterial stiffness [7]. However, PWV measurements are much more complicated to perform, rely on external inaccurate measurements of distances between the pulse wave recording sites, and can bring a large discomfort to a patient [9]. Here, we propose the framework in a modern spirit of highly personalized medicine that can bridge the gap between those two methods as it can provide, among others, the information about arterial stiffness separately for large and small arteries from a single peripheral waveform measurement.

Proposed framework correctly identified increased arterial stiffness in hemodialysis patients, with its transient decrease during the treatment session. Those changes in the arterial stiffness translate to hemodialysis induced decrease in model-predicted pulse wave velocity, the effect that has been observed in other clinical studies [36]. Detected hemodialysis-induced changes in some of the framework-derived and standard PWA-derived indices can be directly related to physical changes in blood circulation during treatment session. Namely, initiation of hemodialysis session results in decreased pressure and workload for the heart as the blood volume circulating in the body is reduced by the blood volume required to fill the tubing together with dialyzer itself and because there is an additional (artificial) blood pump for extracorporeal circulation. Further reduction of circulating blood volume during dialysis session due to the removal of excess fluid yields further improvement of heart workload, with the best conditions before the end of the session (assuming the stability of circulatory system and no hypotensive episodes). The final phase of dialysis—the return of the blood from the extracorporeal circuit to the body circulation and switching off the blood pump—has a reverse effect on the heart and the pulse wave: the changes are towards pre-dialytic conditions. Our goal at the study onset was also to check what is the influence of the applanation tonometry done only in the arm without the fistula. The investigator that was performing the measurements did initially try to capture the radial waveform at the arm with the arteriovenous (AV) fistula, but unfortunately, due to a weak pulse, it was impossible in most cases to obtain recording with sufficient quality.

One of the possible limitations of our study could be the existence of AV fistula in hemodialysis patients which may influence pulse wave-derived parameters, both from the standard PWA method and when using the proposed model. This is because the AV fistula, with the blood flows that may exceed 600 ml/min, substantially alters the normal systemic blood flow and results in the increased cardiac output [37]. To estimate the potential effect of the AV presence on the obtained results we introduced the AV fistula into the model following the work by Huberts et al. [38, 39]; see S1 File for details. Comparison of the simulated aortic and radial pressure waveforms before and after introduction of arteriovenous fistula showed that AV creation results in decreased systemic pressure without substantial changes in the pulse wave shape; see S2A and S2B Fig. Most importantly, this decrease in systemic pressure was obtained for clinically relevant flows through AV fistula, see S2C Fig. Further investigations of the radial-to-aortic transfer function showed that introduction of AV fistula has a little effect on its shape, see S3 Fig, indicating that PWA method is not affected by the presence of vascular access. To check whether it is valid to use the model without fistula for hemodialysis patients, as it has been done in this work, we performed model fitting to the profiles simulated using the model with AV fistula. Results of the parameter estimation showed that using the model without fistula introduces small approximation error, see S4 Fig, and can result in underestimation of small artery stiffness (parameter *k*_1_) and scaling of terminal resistances (parameter *S*_*R*_), see S5 Fig. Therefore, because we show increased arterial stiffness, our initial assumption to use the model without fistula, in order to keep the number of patient- and group-specific parameters to minimum, does not corrupt the results of our study.

Another possible limitation of our study is that the parameter estimating procedure simultaneously adjusts only six patient-specific parameters when trying to minimize the discrepancy between the model-predicted and measured peripheral artery waveform. Of course there are other parameters in the model that are currently fixed at the literature values and thus, are not patient-specific. This is because there is only a limited amount of information present in the peripheral pressure profile recording what does not allow us to robustly estimate all of the model parameters nor the exact shape of the heart ejection profile. Recorded pressure profiles have multiple characteristic points that differ significantly between the subjects (see five randomly selected recorded pressure profiles in S6 Fig) allowing us to identify a specific subset of parameters chosen in the current work. Of course we cannot guarantee the uniqueness of those selected parameters, but for each subject we tried to extensively search the whole possible parameter space by starting first with heuristic particle swarm optimization (PSO) algorithm coupled with a gradient based procedure; see [20] for further details.

In conclusion, we showed that patient-specific pulse wave propagation modeling coupled with radial pressure recording can correctly identify increased arterial stiffness in HD patients, while regular PWA-based biomarkers failed to show significant differences. However, further model testing in larger populations and investigating other biomarkers is needed to confirm these findings. It is worth mentioning, that PWA is being nowadays routinely performed in many hospitals and thus, our framework can shed a new light on those existing datasets.

## Supporting information

S1 File Description of the model with upper and lower arm arteriovenous fistula. (PDF)

S1 Fig Interdependence of the clinical and pulse wave-derived characteristics in healthy individuals. Color coded are the Spearman correlation coefficients for four different pulse wave measurement moments. Font color encodes the type of the parameter, i.e. whether it is model-derived (red), SphygoCor derived (black), or it is a basic clinical characteristic (blue). White squares indicates lack of statistical significance. Three black squares highlight the main correlation clusters.
(TIF)

S2 Fig Impact of the arteriovenous fistula on the model-predicted pressure profiles. AV fistula was introduced into the modeled arterial tree for each individual from the control group. Average peripheral (A) and aortic (B) blood pressure profiles show that creation of AV fistula results mostly in decreased pressure values while the shape of the wave remains similar. (C) Model-predicted flows through fistula introduced in lower or upper arm. Each box represents the interquartile range with the red line being the sample median. Red + signs denote outliers, i.e. values that are more than 1.5 times the interquartile range away from the top or bottom of the box. Whiskers show the furthest observations when neglecting outliers. Black line with an asterisk above indicates p-value < 0.05.
(TIF)

S3 Fig Impact of the arteriovenous fistula on radial-to-aortic transfer function. For each individual from the control group we calculated the radial-to-aortic transfer function before and after AV fistula creation. Shown is the average modulus and phase of the transfer function in the frequency domain.
(TIF)

S4 Fig Ability of the fistula-free model to approximate the profiles generated by the model with upper or lower arm fistula. The model considered in the main text of the manuscript, i.e. the model without fistula, was fitted to radial pressure profiles generated by the model with AV fistula for each individual from control population. Shown are boxplots of obtained average relative errors. No statistical difference between lower and upper arm fistula was detected.
(TIF)

S5 Fig Changes in estimated parameters values when fitting the model without fistula to the profiles generated by the model with incorporated fistula. Shown are percent changes between true parameters, i.e. those used for generating radial profiles from the model with AV fistula, and those estimated by fistula-free model. Asterisks indicate statistical significance with p-value < 0.05.
(TIF)

S6 Fig Five randomly selected recorded radial pressure profiles. (TIF)
