# Supplementary material for: Patient-specific pulse wave propagation model identifies cardiovascular risk characteristics in hemodialysis patients
Source: PLoS Comput Biol. 2018 Sep 14;14(9):e1006417. doi: 10.1371/journal.pcbi.1006417 (PMC6157900; doi:10.1371/journal.pcbi.1006417)
Supplement: S1 File — (PDF) [file pcbi.1006417.s001.pdf]

S1 File. Description of the model with upper and lower arm arteriovenous fistula.

Supplementary Materials to the article "Patient-specific pulse wave propagation model identifies cardiovascular risk characteristics in hemodialysis patients" by J. Poleszczuk et. al.

To estimate the potential effect of the arteriovenous (AV) fistula presence on the obtained results we introduced the arteriovenous fistula into the model following the work by Huberts et al. (1, 2). To this end we considered two scenarios: upper and lower arm arteriovenous fistula. The former was modeled by introducing another vascular segment branching 30 cm from the left brachial artery inlet and having the following dimensions: length L = 40 cm, inlet and outlet radii equal to 0.25 and 0.13 cm, respectively. Brachial artery just before and just after the bifurcation point had the radius of 0.3 and 0.27 cm, respectively. We assumed that the lower arm fistula has exactly the same reference dimensions as the one in the upper arm, but it branches 10 cm from the inlet of radial artery with the radial artery having the radius of 0.17 cm both just before and just after the bifurcation point.

In addition to the geometry one needs to define the inflow and outflow conditions for the fistula segment. The definition of the later is simpler than in the case of the normal arterial segment as the AV fistula is connected directly to the venous system and thus, we don't need to consider the resistances and compliances of the capillary beds. In other words, we assume a constant pressure condition at the AV fistula outlet that is equal to the pressure at the venous end of the arterial tree (15 mmHg as in our previous study (3)). Much more problematic is the condition at the inlet to the AV fistula, because the branching angle, i.e. angle between the daughter arteries, is obtuse and we can no longer assume pressure equality as in the other branching points; compare schematic representation of the branching point in Fig. 1.

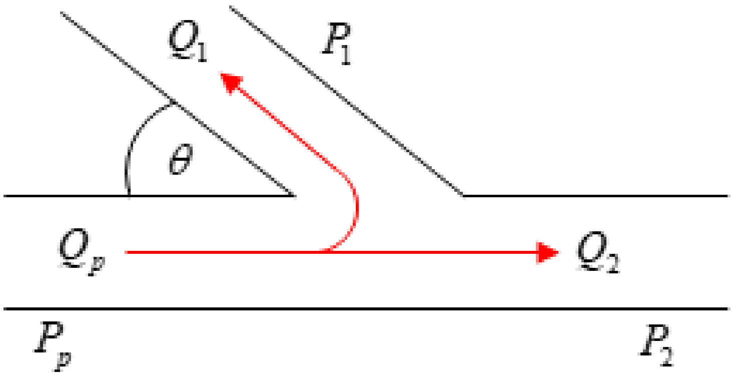

Figure 1. Schematics of the branching point for the arteriovenous fistula. Subscript *p* indicates parent vessel (brachial and radial artery for upper and lower arm fistula respectively) and fistula is the branch labeled with subscript 1. P and Q are the pressure and flow, respectively.

Of course we still assume no leak condition, i.e. that the outflow from the parent vessel is balanced by the inflow to the daughter vessels:

$$Q_p(t,L) = Q_1(t,0) + Q_2(t,0) \quad (1.1)$$

Following the work by Huberts et al. (1, 2) we assume the following equations governing the difference between pressures at the outlet of the parent vessel and inlets to the daughter vessels:

$$P_p(t,L) - P_i(t,0) = K_i \frac{1}{2} \rho v_p^2(t,L), \quad i = 1,2 \quad (1.2)$$

where ρ is the blood density and $v_p(t,L) = Q_p(t,L) / A_p(t,L)$ is the averaged cross-sectional blood velocity (A is the cross-sectional area). What is most important, coefficients K_i are not constant, but depend on the unbalance between the flows and the cross-sectional areas, i.e.

$$K_1 = k_1 (1-\bar{q})^2 + \bar{q}^2 \left[k_2 \cot\left(\frac{\theta}{2}\right) - k_3 + \frac{k_4 - k_5 \bar{A}}{\bar{A}^2} \right] + k_6 \bar{q} (1-\bar{q}) \left(1 + \frac{1}{\bar{A}}\right) \cot\left(\frac{\theta}{2}\right) \quad (1.3)$$

and

$$K_2 = k_7 (1-\bar{q})^2 + k_8 \bar{q}^2 - k_9 \bar{q} (1-\bar{q}), \quad (1.4)$$

where $\theta = 45\deg$ is the angle between fistula segment and the parent artery (compare Fig. 1), $\bar{q}(t,L) = \dfrac{Q_1(t,L)}{Q_p(t,L)}$, $\bar{A}(t,L) = \dfrac{A_1(t,L)}{A_p(t,L)}$, k_1 = 0.95, k_2 = 1.3, k_3 = 0.3, k_4 = 0.4, k_5 = 0.1, k_6 = 0.4, k_7 = 0.03, k_8 = 0.35, and k_9 = 0.2 (1, 2).

References

1. **Huberts W**, **Bode AS**, **Kroon W**, **Planken RN**, **Tordoir JH**, **van de Vosse FN**, **Bosboom EM**. A pulse wave propagation model to support decision-making in vascular access planning in the clinic. *Med Eng Phys* 34: 233–248, 2012.

2. **Huberts W**, **de Jonge C**, **van der Linden WP**, **Inda MA**, **Passera K**, **Tordoir JH**, **van de Vosse FN**, **Bosboom EM**. A sensitivity analysis of a personalized pulse wave propagation model for arteriovenous fistula surgery. Part B: Identification of possible generic model parameters. *Med Eng Phys* 35: 827–837, 2013.

3. **Poleszczuk J**, **Debowska M**, **Dabrowski W**, **Wojcik-Zaluska A**, **Zaluska W**, **Waniewski J**. Subject-specific pulse wave propagation modeling: Towards enhancement of cardiovascular assessment methods. *PLoS One* 13: e0190972, 2018.
