# Supplementary figures and images for: Patient-specific pulse wave propagation model identifies cardiovascular risk characteristics in hemodialysis patients
Source: PLoS Comput Biol. 2018 Sep 14;14(9):e1006417. doi: 10.1371/journal.pcbi.1006417 (PMC6157900; doi:10.1371/journal.pcbi.1006417)

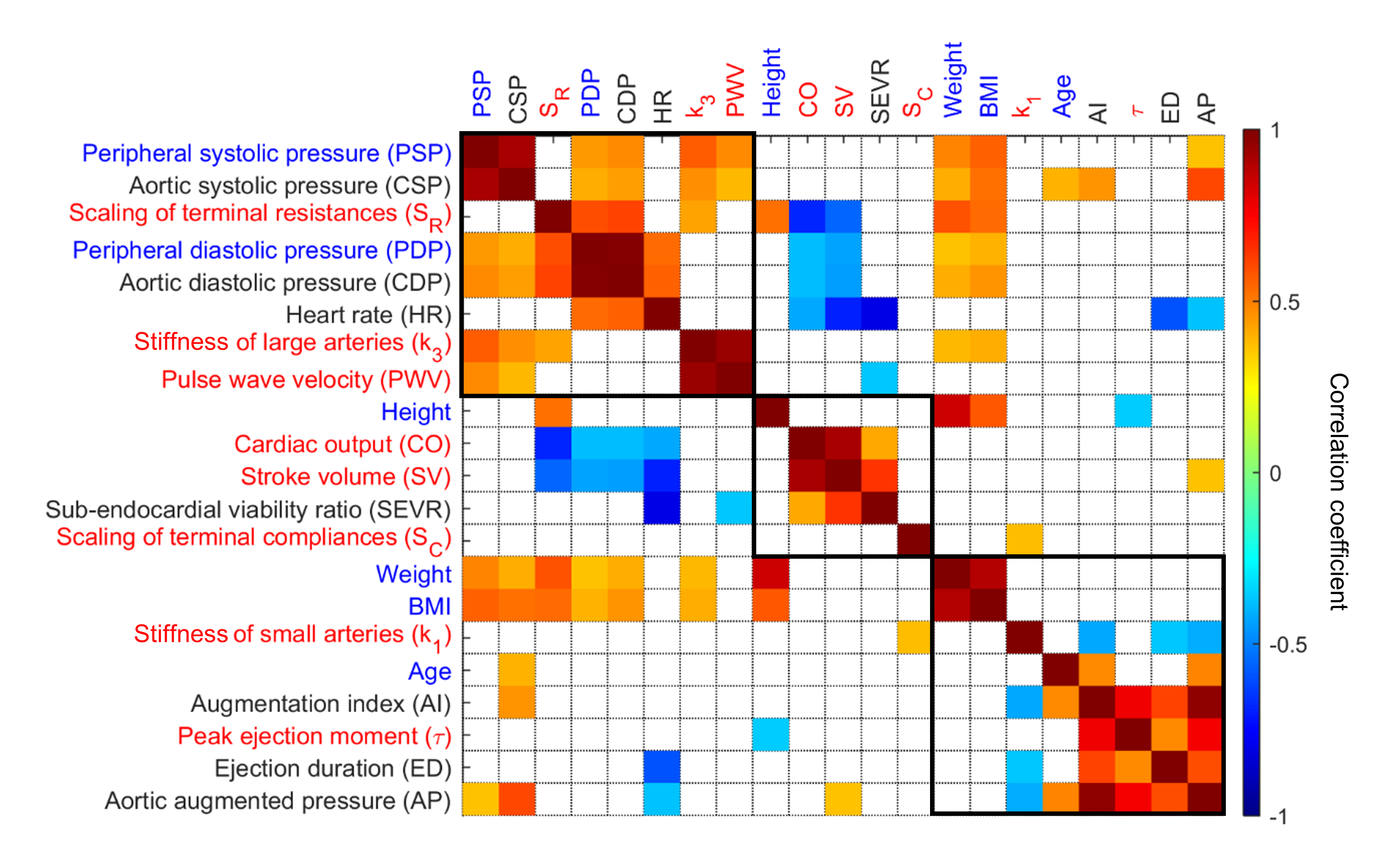

Supplement: S1 Fig — Color coded are the Spearman correlation coefficients for four different pulse wave measurement moments. Font color encodes the type of the parameter, i.e. whether it is model-derived (red), SphygoCor derived (black), or it is a basic clinical characteristic (blue). White squares indicates lack of statistical significance. Three black squares highlight the main correlation clusters. (TIF) [file pcbi.1006417.s002.tif]

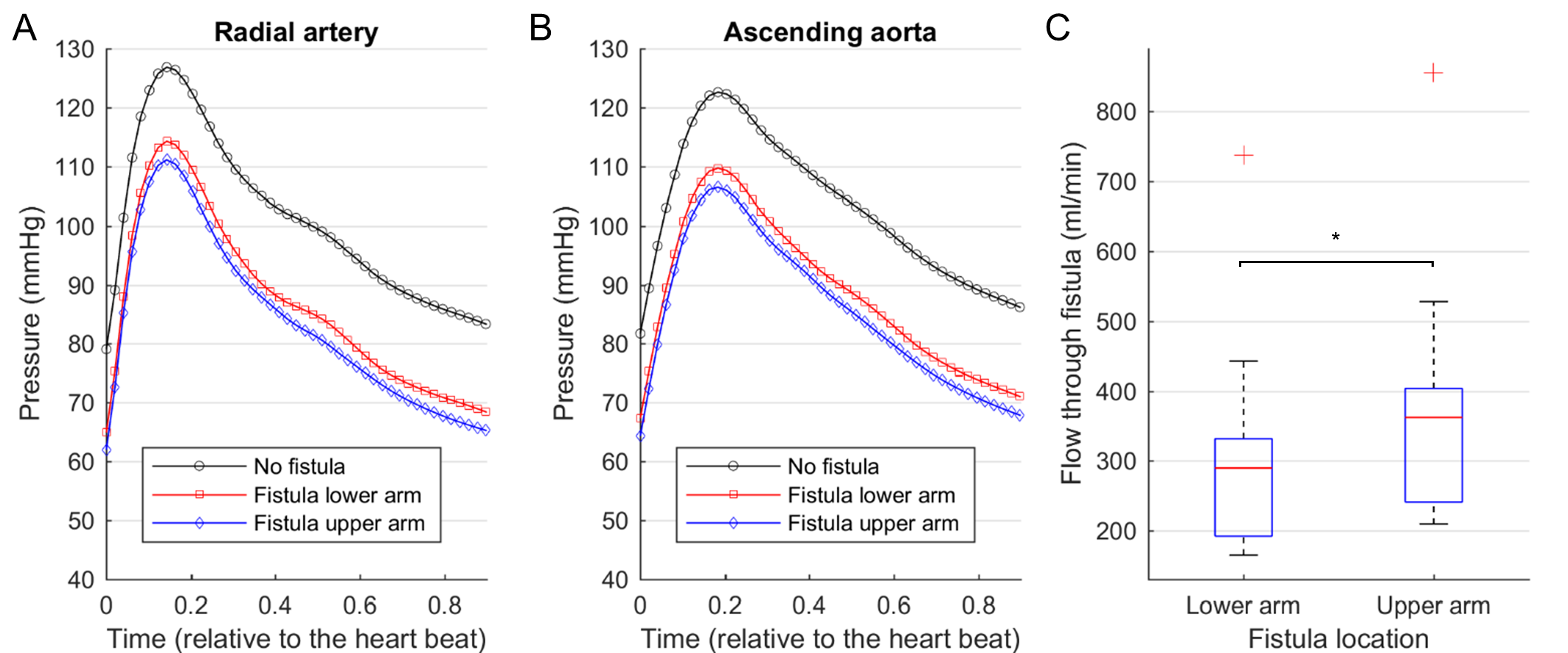

Supplement: S2 Fig — AV fistula was introduced into the modeled arterial tree for each individual from the control group. Average peripheral (A) and aortic (B) blood pressure profiles show that creation of AV fistula results mostly in decreased pressure values while the shape of the wave remains similar. (C) Model-predicted flows through fistula introduced in lower or upper arm. Each box represents the interquartile range with the red line being the sample median. Red + signs denote outliers, i.e. values that are more than 1.5 times the interquartile range away from the top or bottom of the box. Whiskers show the furthest observations when neglecting outliers. Black line with an asterisk above indicates p-value < 0.05. (TIF) [file pcbi.1006417.s003.tif]

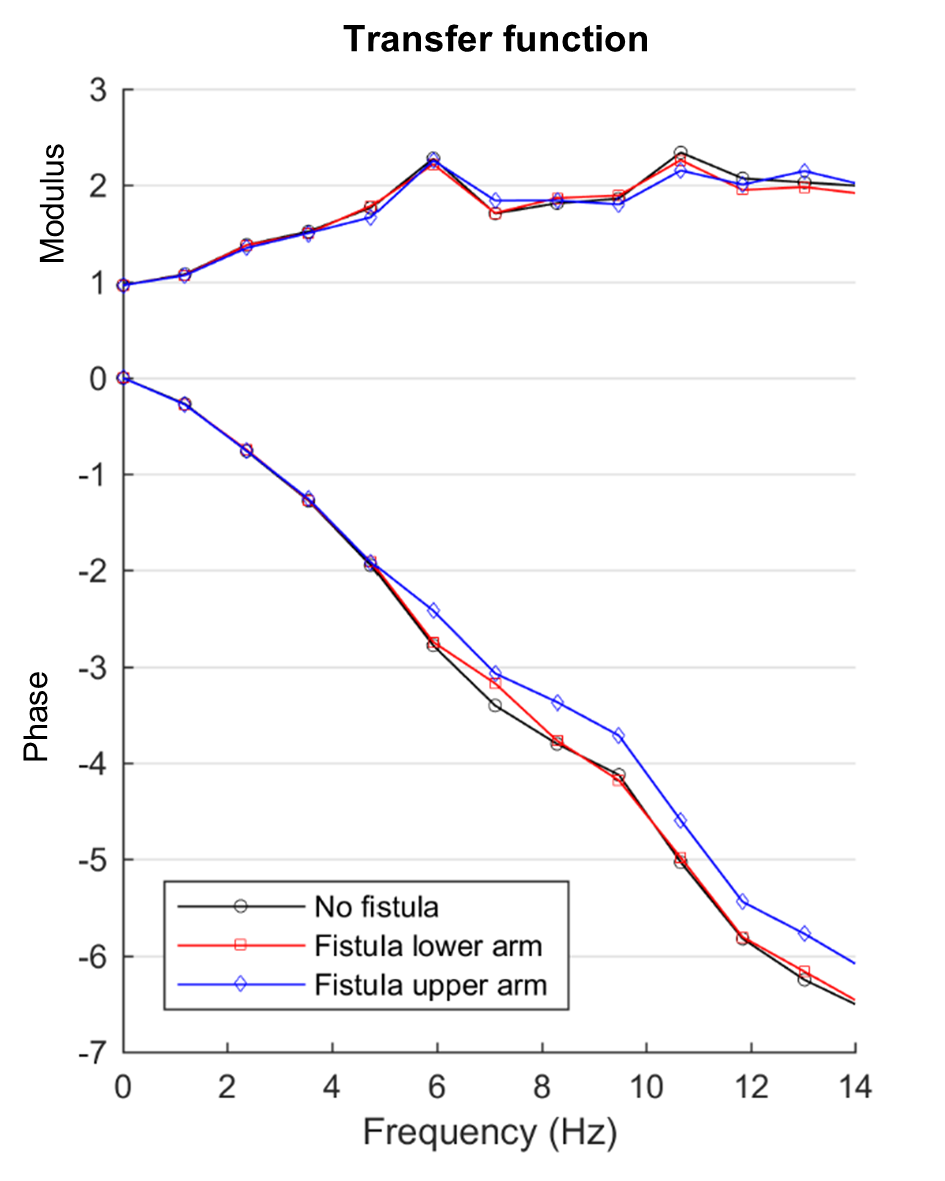

Supplement: S3 Fig — For each individual from the control group we calculated the radial-to-aortic transfer function before and after AV fistula creation. Shown is the average modulus and phase of the transfer function in the frequency domain. (TIF) [file pcbi.1006417.s004.tif]

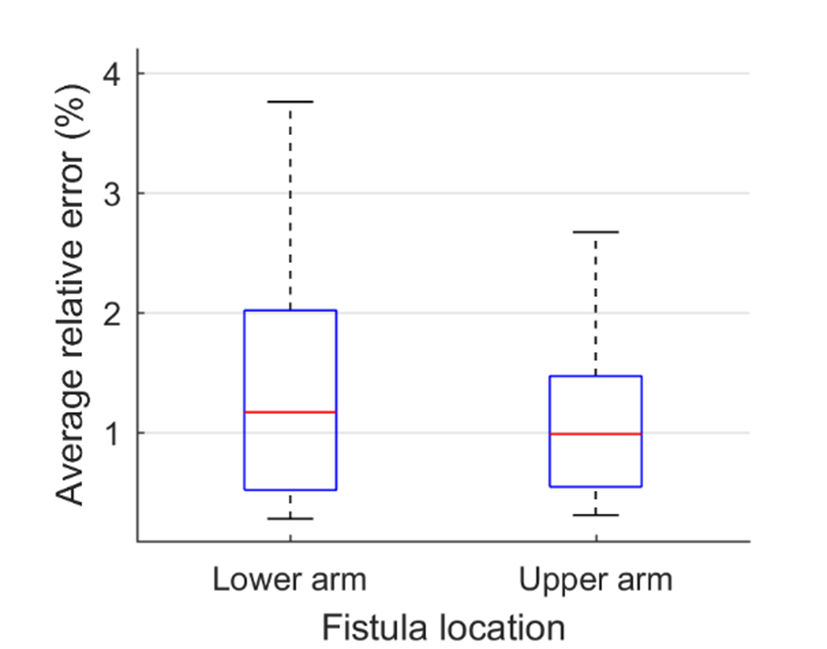

Supplement: S4 Fig — The model considered in the main text of the manuscript, i.e. the model without fistula, was fitted to radial pressure profiles generated by the model with AV fistula for each individual from control population. Shown are boxplots of obtained average relative errors. No statistical difference between lower and upper arm fistula was detected. (TIF) [file pcbi.1006417.s005.tif]

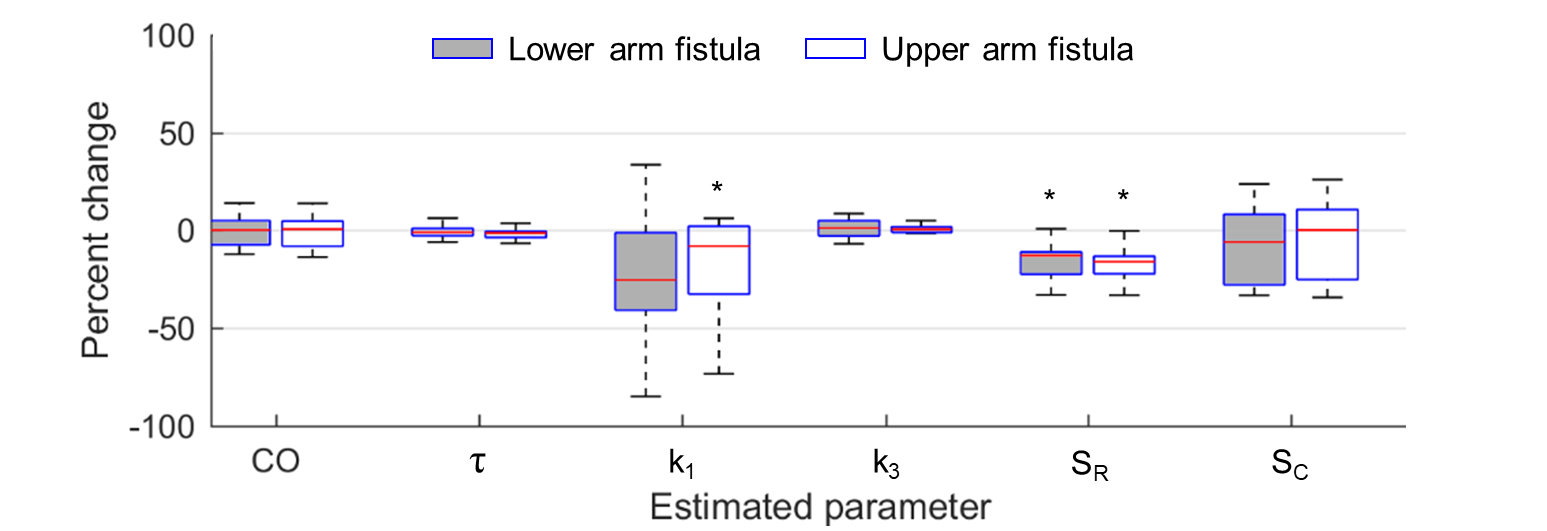

Supplement: S5 Fig — Shown are percent changes between true parameters, i.e. those used for generating radial profiles from the model with AV fistula, and those estimated by fistula-free model. Asterisks indicate statistical significance with p-value < 0.05. (TIF) [file pcbi.1006417.s006.tif]

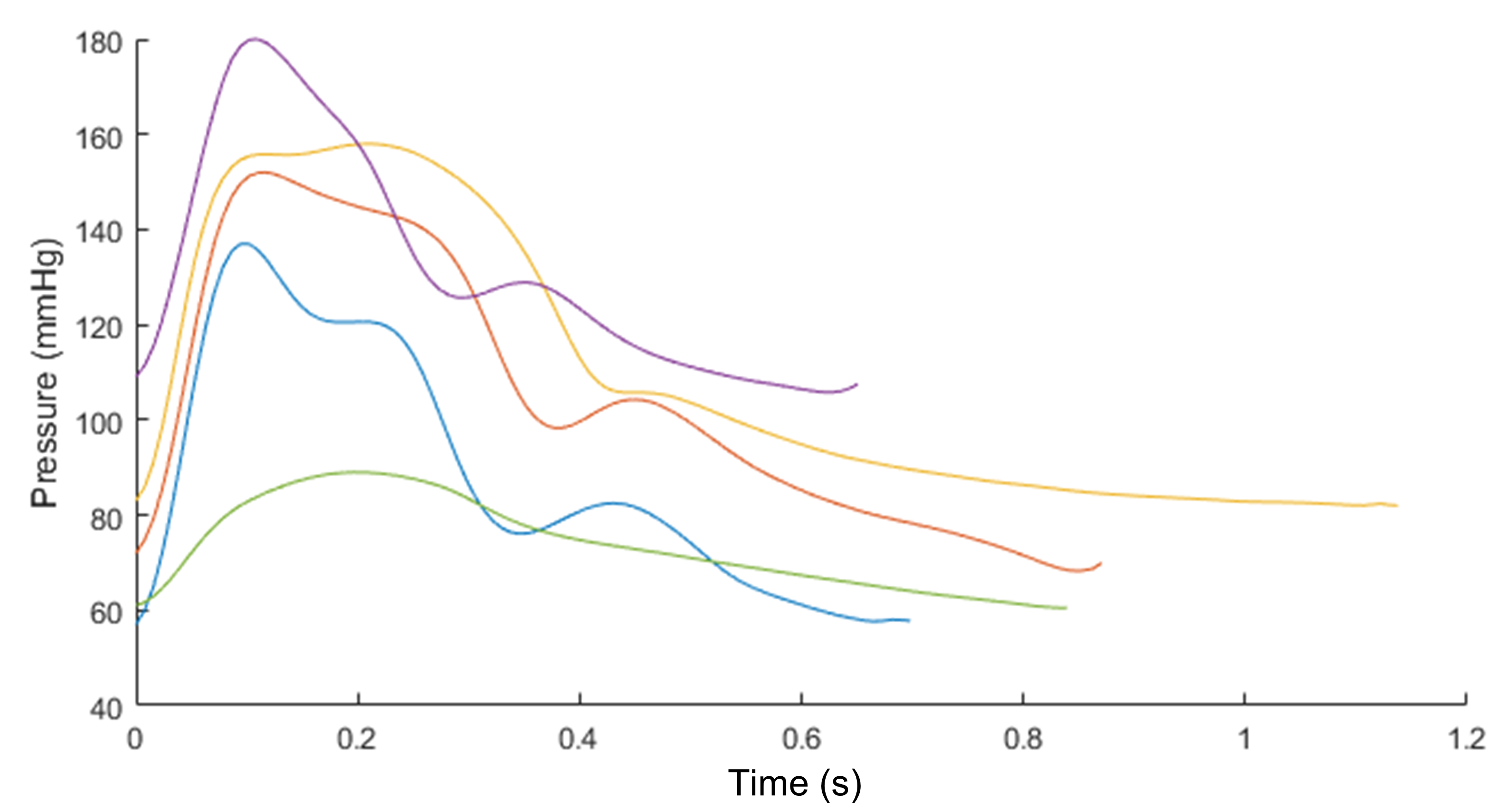

Supplement: S6 Fig — (TIF) [file pcbi.1006417.s007.tif]
